# Efficacy and safety of bloodletting for herpes zoster: A protocol for systematic review and meta-analysis

**DOI:** 10.1097/MD.0000000000026169

**Published:** 2021-06-04

**Authors:** Xiaoyan Wang, Shaolei Huang, Guoliang Shao, Jian Zhang, Suyao Wang, Yanfei Lv, Feng Dong, Jing Han, Dianhui Yang

**Affiliations:** a Shandong University of Traditional Chinese Medicine; b Affiliated Hospital of Shandong University of Traditional Chinese Medicine; c Jinan Lixia District People's Hospital; d Shandong Provincial Hospital affiliated to Shandong First Medical University, Jinan, Shandong, China.

**Keywords:** bloodletting, herpes zoster, herpesvirus, protocol, systematic review

## Abstract

**Background:**

The study aims to evaluate the effectiveness and safety of bloodletting therapy for herpes zoster.

**Methods:**

The following electronic databases will be searched from PubMed (1966 to March 2020), the Cochrane Central Register of Controlled Trials (update to March 2020), EMBASE (1980 to March 2020), China National Knowledge Infrastructure (1979 to March 2020), Wan Fang Data (1980 to March 2020), Chinese Scientific Journal Database (1989 to March 2020), Chinese Biomedical Database (1978 to March 2020) and traditional Chinese medicine Literature Analysis and Retrieval Database (1949 to March 2020). All randomized controlled trials without any limitation of blinding or publication language about this topic will be included, exclude cohort studies and case reports. Two independent researchers will operate article retrieval, duplication removing, screening, quality evaluation, and data analyses by Review Manager (V.5.3.5). Meta-analyses, subgroup analysis, and/or descriptive analysis will be performed based on the included data conditions.

**Results:**

High-quality synthesis and/or descriptive analysis of current evidence will be provided from cure rate, converting to clinical diagnosis rate, and side effects of bloodletting.

**Conclusion:**

This study will provide the evidence of whether bloodletting is an effective and safe intervention for herpes zoster.

**PROSPERO registration number:**

CRD42020171976

## 1 Introduction

### 1.1 Description of the condition

Herpes zoster is a common dermopathic disease which is caused by reactivation of varicella-zoster virus and it spreads from a single sensory ganglion to the neural tissue and dermatome of the affected segment.^[1,2]^ The characteristic rash and acute pain are typically distressing symptoms which disturb the normal life of patients^[3,4]^ and bring both medical and economic burdens,^[5]^ moreover, it also has side effects, such as chronic pain and Post-herpetic neuralgia (PHN) which refers to pain that remains after the healing of rashes from herpes zoster.^[6]^ Many research shows that the incidence of herpes zoster is increasing,^[7,8]^ herpes zoster is currently treated primarily with antiviral drugs and vaccine, yet this treatment has been debated.^[9–11]^ As more and more treatments for herpes zoster are developed,^[12,13]^ Acupuncture is an effective therapy in treating herpes zoster, besides bloodletting, as a method of acupuncture treatment, is widely used in clinical practice, bloodletting might have an advantage for analgesia and reducing adverse reactions. Recently, many scholars proposed that bloodletting therapy has significant clinical effects in the treatment of herpes zoster,^[14]^ relieving pain and improving patients’ quality of life.^[14,15]^ From literature, we found some clinical trial reports on bloodletting therapy for herpes zoster, but there is no systematic review and meta-analysis about the therapeutic effect of the therapy. Therefore, this review aims to evaluate the beneficial and harmful effects of wet cupping therapy for treatment of herpes zoster.

### 1.2 Description and function of intervention

Bloodletting is an important part of traditional Chinese medicine and has been used as an important complementary therapy in the word. It treats diseases through using a sharp instrument such as trigonometric needle, with or without an auxiliary method like cupping, filiform needle, plum blossom needle, to prick the superficial blood vessels and remove a few drops of blood from the patient to treat diseases and has the characteristics of precise efficacy and non-toxic side effects. Bloodletting therapies include many different treatments, such as blood-pricking therapy,^[13]^ pricking collateral and bloodletting therapy, cupping and bloodletting,^[16,17]^ auricular acupuncture and bloodletting therapy, auricle cutting method,^[18]^ cutaneous needle tapping or skin needle pricking,^[19]^ fire needle,^[20–22]^ etc. Bloodletting therapy has been proved effective in some skin diseases and is of great significance for the herpes zoster.

### 1.3 Why the review is important

According to the published research, there is a lack of high quality evidence on bloodletting in the treatment of herpes zoster. Therefore, this systematic review aims to assess the effectiveness and safety of bloodletting therapy for herpes zoster.

## 2 Methods

This systematic review protocol has been registered in the PROSPERO network (No. CRD42020171976). All steps of this systematic review will be performed according to the Cochrane Handbook (5.2.0).

### 2.1 Selection criteria

#### 2.1.1 Types of studies

Randomized controlled trials (RCTs) of bloodletting therapy for herpes zoster without any limitation of blinding or publication language will be included. RCTs that involve bloodletting used alone or in combination with other routine treatments will be included. The studies of bloodletting therapy combined with a different type of traditional Chinese medicine (TCM) therapy (e.g., Chinese herb decoction, acupuncture and other therapies) will be excluded.

#### 2.1.2 Types of patients

Patients who were diagnosed as herpes zoster will be included, without limits on gender, age, illness course or race.

#### 2.1.3 Types of interventions and comparisons

Interventions can be bloodletting therapy used alone or in combination with other routine treatments. Multiple control interventions will be included: no treatment, placebo, drug control (e.g., antiviral drugs, nutritional neuromedicine) and other interventions (e.g., standard care, drugs, Chinese medicine). Comparisons contain bloodletting therapy combined with a different type of TCM therapy will be excluded. Interventions of bloodletting combined with other routine treatments will also be included, only if the other therapies were used as comparisons.

#### 2.1.4 Types of outcomes

The primary outcome in this study was pain intensity, as measured by the Visual Analog Scale (VAS), Numerical Rating Scale, McGill Pain Questionnaire, Verbal Rating Scale as well as several other scales for measuring pain. VAS is the most common measurement to assess pain intensity. It is scored on a range of either 0–10 or 0–100. Numerical Rating Scale (0 = no pain, 10 = worst pain) and Verbal Rating Scale (none/very mild/mild-moderate/severe/very severe) are similar to VAS in that pain intensity is measured by numbers and descriptions. A higher score indicates greater pain intensity. The McGill Pain Questionnaire consists of 20 subcategories, in four parts, to measure pain properties and intensity. A higher score means more serious pain.

The secondary outcome included onset of pain relief time, blisters disappear and scabs form time, life quality, incidence of PHN and adverse effects. The onset of pain relief time is the amount of time before pain relief began. The shorter the time requirement, the more effective the treatment. The HAMA consists of 14 items ranging from 0 to 4, which assess the severity of patients’ anxiety states. A higher score suggests a higher degree of anxiety. In this study, QOL was an assessment method for QOL related to pain, with a range of 0 to 10. A higher score meant better QOL.

### 2.2 Search methods for identification of studies

#### 2.2.1 Electronic searches

The following electronic databases will be searched from PubMed (1966 to March 2020), the Cochrane Central Register of Controlled Trials (update to March 2020), EMBASE (1980 to March 2020), China National Knowledge Infrastructure (1979 to March 2020), Wan Fang Data (1980 to March 2020), Chinese Scientific Journal Database (1989 to March 2020), Chinese Biomedical Database (1978 to March 2020) and TCM Literature Analysis and Retrieval Database (1949 to March 2020). RCTs written in English and Chinese will be eligible for inclusion. Exemplary search strategy of PUBMED is listed in Table 1. According to the difference of databases, a combination of keywords will be performed, including: ‘bloodletting,’ ‘herpes zoster,’ ‘randomized,’ ‘trial,’ et al.

**Table 1:** PUBMED search strategy.

Number	Search terms
#1	MeSH Major Topic: herpes zoster
#2	MeSH Major Topic: Herpesvirus
#3	MeSH Major Topic: PHN
#4	MeSH Major Topic: Varicella-zoster virus (VZV)
#5	MeSH Major Topic: sequela
#6	MeSH Major: neuralgia
#7	MeSH Major: snake string sore
#8	MeSH Major: bilateral herpes zoster
#9	MeSH Major: shingles
#10	MeSH Major: bloodletting therapy
#11	MeSH Major Topic: blood-pricking therapy
#12	MeSH Major Topic: cupping and bloodletting
#13	MeSH Major Topic: plum -blossom needle therapy
#14	MeSH Major Topic: trigonometric needle
#15	MeSH Major Topic: auricle cutting method
#16	MeSH Major Topic: fire needle
#17	MeSH Major Topic: cutaneous needle tapping
#18	MeSH Major Topic: filiform needle
#19	MeSH Major Topic: pricking collateral and bloodletting therapy
#20	MeSH Major Topic: auricular acupuncture and bloodletting therapy
#21	MeSH Major Topic: skin needle pricking
#22	MeSH Major Topic: infants
#23	MeSH Major Topic: children
#24	MeSH Major Topic: pediatric
#25	MeSH Major Topic: adult
#26	MeSH Major Topic: elderly
#27	MeSH Major Topic: agedness
#28	MeSH Major Topic: gerontism
#29	#2 or #3 or #4 or#5 or #6 or #7 or #8 or #9
#30	#10 or #11 or #12 or #13 or #14 or #15 or #16or #17 or #18 or #19 or #20 or #21
#31	or #22 or #23 or #24 or #25 or #26 or #27or#28
#32	#1 and #29 and #30 and #31

#### 2.2.2 Searching other resources

At the same time, we will retrieve other resources to complete the deficiencies of the electronic databases, mainly searching for the clinical trial registries and grey literature about bloodletting for herpes zoster on the corresponding website.

### 2.3 Data collection and analysis

#### 2.3.1 Selection of studies

**Figure 1:**
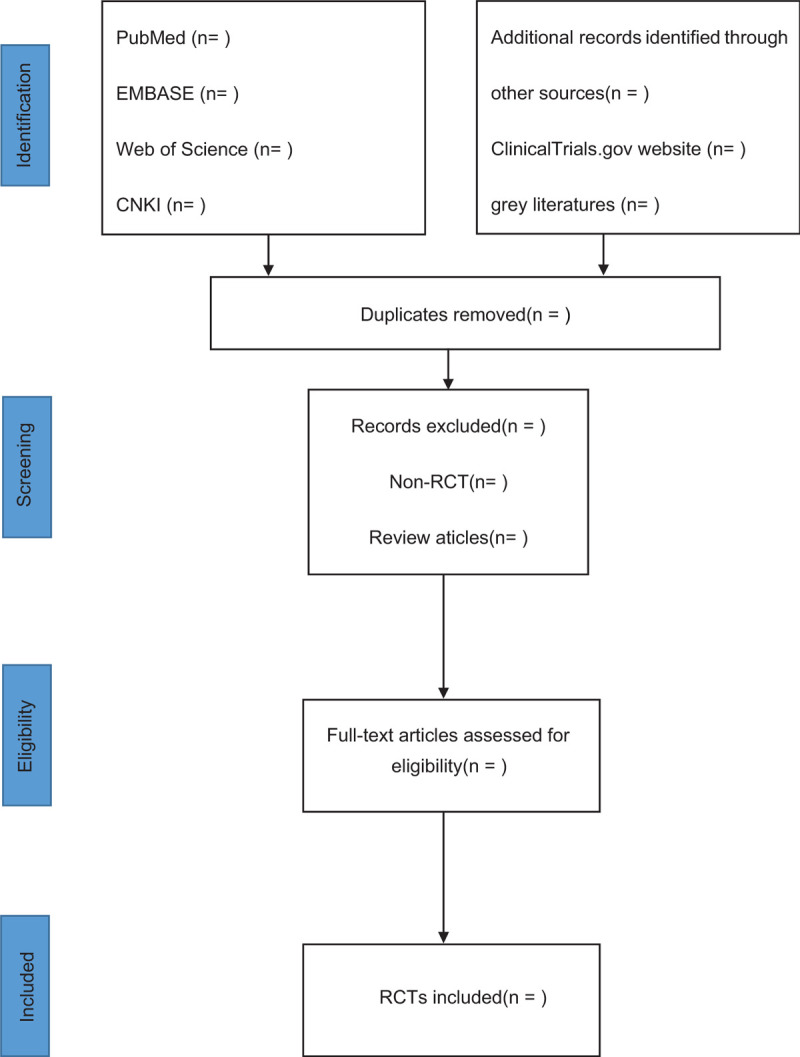
Study flowchart of selected articles for final analysis.

Two reviewers (SLH and XYW) will independently select the studies. They will check the results with each other. When disagreements occur, a third reviewer (GLS) will make the final decision. They will read the full texts of all included studies if necessary. Screening operation will flow the diagram of Figure 1. If the full literatures are unable to be obtained or related data are incomplete, we will contact the corresponding author.

#### 2.3.2 Assessment and quality of included studies

Two reviewers (JH and SYW) will evaluate quality of included articles and assess the risk of bias based on Cochrane Handbook 5.2.0^[23]^ The following seven items, such as random sequence generation, allocation concealment, blinding of participants and personnel, blinding of outcome assessment, incomplete outcome data, selective outcome reporting, and other bias, are evaluated by three grades of “low bias,” “high bias,” and “unclear bias.” Divergence of evaluation will also consult a third reviewer (DHY).

#### 2.3.3 Data extraction

Two independent reviewers (SLH and YFL) will extract data after selection and quality assessment; they will extract the data using a standardized data extraction form and any differences of opinion between them will be resolved through discussion; if failed, they will discuss with the third reviewer (JZ). Data will be recorded onto an electronic form, including the basic information of the article (the title of article, first author, year, and language), inclusion and exclusion criteria, the baseline of the study (the sample size, sex ratio, age and course), interventions in the observation group and the control group, and outcome measures.

#### 2.3.4 Measures of treatment effect

Two reviewers (XYW and FD) will perform analysis independently and then cross-check treatment effect with Review Manager 5.3.5. The count data will be represented using relative risk, while continuous variables will be represented by standard mean differences (MD), and for both, a 95% confidence interval will be calculated. If there is heterogeneity between the interventions (I is greater than 50%), the stochastic effect model is used for calculation; otherwise, the fixed effect model is used.

#### 2.3.5 Dealing with missing data

Due to the possibility of data loss in the literature, we will contact the corresponding author by email or other means. If the missing data are not available, we will analyze the existing data assumed to be lost at random.

#### 2.3.6 Assessment of heterogeneity

The heterogeneity of studies will be evaluated by I^2^ statistic with RevMan5.3 software. The dichotomous data is represented by risk ratio, continuous data is expressed by MD or standard MD. If there is no heterogeneity (*I*^2^ < 50%, *P* > .001), the data are synthesized using a fixed effect model. Otherwise (50≤I2 < 75%, *P* < .001), a random effect model is used to analyze. if the heterogeneity is significantly high (I2≥75, *P* < .001)), subgroup analysis or descriptive analysis will be performed.

#### 2.3.7 Assessment of reporting bias

Publication bias and other reporting bias will be assessed by creating funnel plots.^[24]^ A symmetrical funnel plot indicates a low risk of bias, while an asymmetric funnel plot indicates a high risk of bias.

#### 2.3.8 Subgroup analysis

Subgroup analysis will be performed based on the results of data synthesis, if adequate studies are available in each group. And if heterogeneity is found to be caused by the specific characteristics of the included study (e.g., course, age, gender the intervention methods, and the measurement methods used in the clinical trials), subgroup analysis will be conducted relevant to these categories.

## 3 Discussion

Herpes zoster causes significant suffering owing to acute and chronic pain or (PHN). herpesvirus-induced neuronal destruction and inflammation causes the principal problems of pain, interference with activities of daily living, and reduced quality of life in the patients.^[25]^ It could bring great threat to public and society. Bloodletting therapy is a kind of important traditional Chinese medicine treatment with simple operation and low cost. China's guidelines for treatment of PHN recommend bloodletting because of its clinical effects.^[26]^ Lots of Chinese hospitals are using bloodletting therapy to treat herpes zoster. If the evidence could prove bloodletting is useful for herpes zoster, it might save many costs and be beneficial to worldwide people. However, no systematic reviews on this topic have been published. In order to give compelling evidence and better guide in clinic practice, all actions of this review will be performed according to Cochrane Handbook 5.2.0.

## Author contributions

**Conceptualization:** Xiaoyan Wang, Dianhui Yang.

**Data curation:** Xiaoyan Wang, Shaolei Huang, Yanfei Lv, Jing Han, Dianhui Yang.

**Formal analysis:** Shaolei Huang, Guoliang Shao, Jing Han.

**Funding acquisition:** Dianhui Yang.

**Investigation:** Suyao Wang, Jing Han.

**Methodology:** Guoliang Shao.

**Project administration:** Jian Zhang.

**Resources:** Xiaoyan Wang, Guoliang Shao, Suyao Wang, Jian Zhang, Feng Dong.

**Validation:** Yanfei Lv.

**Visualization:** Yanfei Lv, Feng Dong.

**Writing – original draft:** Xiaoyan Wang, Shaolei Huang, Dianhui Yang.

**Writing – review & editing:** Xiaoyan Wang.

## References

[1] Schmader K. Herpes Zoster. Ann Intern Med 2018;169:ITC19–31. 30083718 10.7326/AITC201808070

[2] Gross G Schöfer H Wassilew S. Herpes zoster guideline of the German Dermatology Society (DDG). J Clin Virol 2003;26:277–93. 12637076 10.1016/s1386-6532(03)00005-2

[3] García-González AI Rosas-Carrasco O. Herpes zoster and post-herpetic neuralgia in the elderly: Particularities in prevention, diagnosis, and treatment. Gac Med Mex 2017;153:92–101. 28128811

[4] Gater A Abetz-Webb L Carroll S Mannan A Serpell M Johnson R. Johnson R. Burden of herpes zoster in the UK: findings from the zoster quality of life (ZQOL) study. BMC Infect Dis 2014;14:402. 25038799 10.1186/1471-2334-14-402 PMC4223600

[5] Lim HW. The burden of skin disease in the United States. J Am Acad Dermatol 2017;76:958–72. 28259441 10.1016/j.jaad.2016.12.043

[6] Schmader K. Herpes Zoster. Clin Geriatr Med 2016;32:539–53. 27394022 10.1016/j.cger.2016.02.011

[7] Kawai K. Increasing incidence of herpes zoster over a 60-year period from a population-based study. Clin Infect Dis 2016;63:221–6. 27161774 10.1093/cid/ciw296 PMC4928389

[8] Yawn BP. A population-based study of the incidence and complication rates of herpes zoster before zoster vaccine introduction. Mayo Clin Proc 2007;82:1341–9. 17976353 10.4065/82.11.1341

[9] Zhang N Liu K. Efficacy and safety of acupuncture and moxibustion for herpes zoster: A protocol for systematic review and network meta analysis. Medicine (Baltimore) 2020;99:e21905. 32899021 10.1097/MD.0000000000021905 PMC7478486

[10] Chen N Li Q Yang J. Antiviral treatment for preventing postherpetic neuralgia. Cochrane Database Syst Rev 2014;CD006866. 24500927 10.1002/14651858.CD006866.pub3 PMC10583132

[11] O’Connor KM Paauw DS. Herpes zoster. Med Clin North Am 2013;97:503–ix. 23809711 10.1016/j.mcna.2013.02.002

[12] Coyle ME Liang H Wang K. Acupuncture plus moxibustion for herpes zoster: a systematic review and meta-analysis of randomized controlled trials. Dermatol Ther 2017;30:e12468. 10.1111/dth.12468 28338265

[13] Zhang YN Ding D Cao F. Clinical efficacy observation on the treatment of early herpes zoster by plum blossom needle blood-letting with laser irradiation. Infectious Disease Information 2019;32:359–61. +365.

[14] Pei W Zeng J Lu L Lin G Ruan J. Is acupuncture an effective postherpetic neuralgia treatment? A systematic review and meta-analysis. J Pain Res 2019;12:2155–65. 31410050 10.2147/JPR.S199950 PMC6643066

[15] Cao H Zhu C Liu J. Wet cupping therapy for treatment of herpes zoster: a systematic review of randomized controlled trials. Altern Ther Health Med 2010;16:48–54. PMC3151529 21280462

[16] WZ Wen S. Clinical observations on the treatment of herpes zoster by bloodletting plus cupping. Shanghai Journal of Acupuncture and Moxibustion 2014;33:135–6.

[17] Cao H Li X Liu J. An updated review of the efficacy of cupping therapy. PLoS One 2012;7:e31793. 22389674 10.1371/journal.pone.0031793 PMC3289625

[18] Yu YD Xu M Wan QH. Effect analysis of auricle cutting method in the treatment of sequela of snake string sore. China Practical Medicine 2020;15:20–3.

[19] Zuo XH Wang ZT Cui WS. Clinical effect of skin needle stabbing combined with blood- letting puncture and cupping for the treatment of neuralgia caused by herpes zoster. Journal of Clinical Acupuncture and Moxibustion 2015;31:20–2.

[20] Qi HC Zhang CL Li SP. Clinical observation on treating acute herpes zoster by fire acupuncture. Clinical Journal of Chinese Medicine 2018;10:109–10.

[21] Zhu RJ Wu MF Zhang HF. Clinical Observation on 30 Cases of Acute Herpes Zoster Treated with Milli-fire Needle. Chinese Journal of Dermatovenereology of Integrated Traditional and Western Medicine 2016;15:99–102.

[22] Zhang Y Liang Z Li S. Fire needle plus cupping for acute herpes zoster: study protocol for a randomized controlled trial. Trials 2020;21:701. 32762718 10.1186/s13063-020-04599-2 PMC7409425

[23] Higgins JP Altman DG Gøtzsche PC. The Cochrane Collaboration's tool for assessing risk of bias in randomised trials. BMJ 2011;343:d5928. 22008217 10.1136/bmj.d5928 PMC3196245

[24] Lau J Ioannidis JP Terrin N Schmid CH Olkin I. The case of the misleading funnel plot. BMJ 2006;333:597–600. 16974018 10.1136/bmj.333.7568.597 PMC1570006

[25] Weinberg JM. Herpes zoster: epidemiology, natural history, and common complications. J Am Acad Dermatol 2007;57: 6 Suppl: S130–5. 18021864 10.1016/j.jaad.2007.08.046

[26] Neuralgia Egftdatop. Chinese experts consensus on diagnosis and treatment of postherpetic neuralgia. Chin J Pain Med 2016;22:161–7.

